# The role of protein kinase C alpha translocation in radiation-induced bystander effect

**DOI:** 10.1038/srep25817

**Published:** 2016-05-11

**Authors:** Zihui Fang, An Xu, Lijun Wu, Tom K. Hei, Mei Hong

**Affiliations:** 1 College of Life Sciences, South China Agricultural University, Guangzhou, 510642, China; 2 Hefei Institute of Physical Science, Chinese Academy of Sciences, Hefei, 230031, China; 3 Center for Radiological Research, College of Physicians & Surgeons, Columbia University, New York, NY10032, USA

## Abstract

Ionizing radiation is a well known human carcinogen. Evidence accumulated over the past decade suggested that extranuclear/extracellular targets and events may also play a critical role in modulating biological responses to ionizing radiation. However, the underlying mechanism(s) of radiation-induced bystander effect is still unclear. In the current study, A_L_ cells were irradiated with alpha particles and responses of bystander cells were investigated. We found out that in bystander A_L_ cells, protein kinase C alpha (PKCα) translocated from cytosol to membrane fraction. Pre-treatment of cells with PKC translocation inhibitor chelerythrine chloride suppressed the induced extracellular signal-regulated kinases (ERK) activity and the increased cyclooxygenase 2 (COX-2) expression as well as the mutagenic effect in bystander cells. Furthermore, tumor necrosis factor alpha (TNFα) was elevated in directly irradiated but not bystander cells; while TNFα receptor 1 (TNFR1) increased in the membrane fraction of bystander cells. Further analysis revealed that PKC activation caused accelerated internalization and recycling of TNFR1. Our data suggested that PKCα translocation may occur as an early event in radiation-induced bystander responses and mediate TNFα-induced signaling pathways that lead to the activation of ERK and up-regulation of COX-2.

A major paradigm shift in radiation biology in the past decade has resulted from the studies of bystander effect^1^,^2^. Radiation-related bystander effect is defined as the induction of biological effects in cells that are not directly traversed by a charged particle but are in close proximity to cells that are, or have received signals from these irradiated cells. In such a scenario, extranuclear and/or extracellular events may also contribute to the final biological consequences of radiation^3^. Bystander effect-induced damages have been clearly established in cell culture systems, and quite a few reports have demonstrated that bystander effect occurs *in viv*o as well^4^. Although bystander effect has been well described, our knowledge on the mechanisms of the process is still limited. Gap junction-mediated cell–cell communications may be important in mediating bystander effect in confluent cultures of either human^5^ or rodent cells^6^, while in subconfluent cultures, it has been shown that reactive oxygen species, nitric oxide, and cytokines such as tumor growth factor beta (TGFβ) are involved^7^,^8^,^9^. Several signaling pathways including the mitogen-activated protein kinases (MAPK) signaling cascade^10^, the NFκB pathway^11^,^12^ and phosphor-inositide-3-kinase (PI3K)-AKT pathway^13^ were demonstrated to mediate the bystander responses.

Protein kinase Cs (PKCs) are serine/threonin kinases that play important regulatory roles in cell cycle progression, differentiation, apoptosis, cytoskeletal remodeling, modulation of ion channels and secretion^14^,^15^. The activation and translocation of PKC in response to radiation has been demonstrated in different cells lines including smooth muscle cell^16^, mouse normal and neoplastic epidermal cells^17^, and rat hepatocytes^18^. Inhibition of PKC was shown to increase cell sensitivity to ionizing radiation, implicating a critical role of PKC in cellular responses to radiation^19^. Study using medium transfer method also indicated that different PKC isoforms including PKC-βII, PKC-θ, PKCα/β translocate from cytosol to the nuclear fraction in bystander as well as irradiated cells^20^. PKCα is a classical form of PKC. It is predominantly cytosolic but translocates to the membrane fraction in response to PKC activator phorbol 12-myristate 13-acetate (PMA)^21^. However, exact subcellular location of PKC differs among cell lines and stimuli. Activation of PKCα has been related with the induction of MAPK, NF-*κ*B, AP-1 and the secretion of TNFα, IL-6, and IL-10^22^.

In the present study, we demonstrated that translocation of PKCα from cytosol to cell membrane is involved in bystander effect induced by α-particles. Inhibition of PKC led to suppression of extracellular signal-regulated kinases (ERK) activity and cyclooxygenase 2 (COX-2) expression as well as a significantly reduced mutagenic effect. Further studies revealed that cytokine TNFα and its receptor TNFR1 may play a role in the process.

## Results

### PKCα translocated from cytosol to membrane in bystander cells

**Figure 1:**
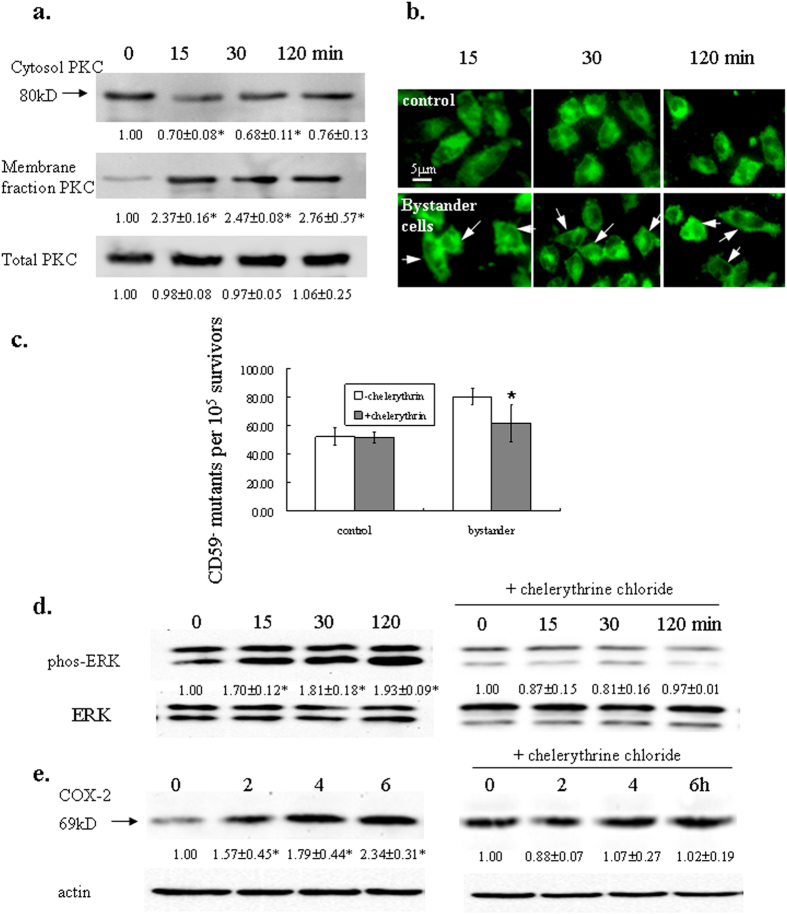
PKC translocation is involved in radiation-induced bystander effect. (**a,b**) PKCα expression in different fractions of bystander A_L_ cells. Cells were irradiated with a 50-cGy dose and cells on the aluminum-wrapped half of the dish were collected at time points indicated. Membrane fraction proteins were extracted and separated with SDS-PAGE, transferred to PVDF membrane and probed with PKCα antibody (**a**). Three independent experiments were performed and a representative blot was shown. Ratios of the corresponding band intensity compared with that of untreated control were measured and calculated with Image J and indicated under each band. Asterisk indicates significant difference between untreated control and the treated groups (*p* < 0.05). In (**b**), cells were fixed with 4% paraformadehyde, probed with PKCα antibody followed by detection with Alexa Fluor 488 secondary antibody. (**c**) Effect of PKC inhibitor chelerythrine chloride on the mutagencity of bystander cells. Exponentially growing A_L_ cells were irradiated as described above. Chelerythrine chloride (10 μM) was added into the cultures 1 hr before irradiation. Data are pooled from four independent experiments. Bars indicate ± S.D. of means. Asterisk indicates significant difference between the treated and untreated control (*p* = 0.03). (d.**e**) Effect of chelerythrine chloride on ERK activity and COX-2 expression. Proteins from bystander cells were extracted with RIPA buffer at the indicated time points after irradiation and probed with phosphor-ERK1/2 or COX-2 antibody. Left panels, untreated controls; right panels, cells were treated with chelerythrine chloride before irradiation. Three independent experiments were performed and a representative blot was shown. Ratios of the corresponding band intensity compared with that of untreated control were measured and calculated with Image J and indicated under each band. Asterisk indicates significant difference between untreated control and the treated groups (*p* < 0.05).

PKC translocation has been implicated in the responses of multiple stimuli including ionizing radiation. It was shown that gamma-radiation could induce apoptosis, growth arrest through activation and translocation of PKCα, PKCε and PKCδ^16^,^23^. To see if PKCα translocates in bystander responses, we investigated PKCα level in different cellular fraction of bystander A_L_ cells. As shown in Fig. 1a, PKCα level in cytosol decreased while its level in the membrane fraction increased significantly in bystander cells, suggesting the responsiveness of PKCα in bystander effect. PKCα level changed as early as 15 min after irradiation and sustained as long as 2 hrs. Immunocytochemistry analysis also showed that PKCα translocates from cytosol to cell membrane in bystander cells. PKCα distributed homogenously before irradiation within A_L_ cells, while its signal intensified along the cell membrane in bystander cells after irradiation (Fig. 1b).

### PKC inhibitor chelerythrine suppressed CD59^−^ mutant fraction in bystander A_L_ cells

To further evaluate the involvement of PKC translocation in bystander effect, we then compared the CD59^−^ mutant yield in bystander A_L_ cells pre-treated with PKC inhibitor chelerythrine chloride and the untreated cells. Chelerythrine is a benzophenanthridine alkaloid that has been shown to inhibit PKC translocation from cytosol to membrane in isolated ileal synaptosomes^24^. As shown in Fig. 1c, there was a 1.5 fold increase in CD59^−^ mutant fraction in bystander A_L_ cells. The pre-treatment of chelerythrine chloride resulted in statistically significant reduction of CD59^−^ mutant yield (*p* = 0.03), which confirmed that PKC translocation is involved in radiation-induced bystander effect.

### Chelerythrine chloride inhibited ERK activation and COX-2 induction

Numerous studies have showed that exposure of cells to ionizing radiation as well as other toxic stresses can induce simultaneous compensatory activation of different MAPK pathways^25^. It was demonstrated that in normal human lung fibroblasts system, both α-irradiated and bystander cells exhibited increased expression of COX-2, and that as an upstream event, up-regulation of phosphor-ERK levels was observed^10^. We also observed an elevated ERK activity and COX-2 expression in bystander A_L_ cells. However, when cells were treated with 10 μM chelerythrine chloride before irradiation, the activation of ERK (Fig. 1d) and induction of COX-2 (Fig. 1e) were suppressed. These results suggested that inhibition of PKC translocation may inhibit bystander responses through the ERK pathway, which in turn attenuated the up-regulation of COX-2. To confirm such an effect was PKCα-specific, we also pretreated A_L_ cells with PKCα-specific inhibitor Gö6976. Gö6976 is a potent and selective PKC inhibitor for conventional PKC isoforms PKCα and β1, with IC50 values of 2.3 nM and 6.2 nM, respectively. We therefore used a concentration of 5 nM to differentiate the effect caused by PKCα or β1. It was found out that 5 nM Gö6976 also suppressed ERK activation and COX-2 expression (Supplementary Fig. S1).

### TNFalpha level increased in directly irradiated cells but not in bystander cells

**Figure 2:**
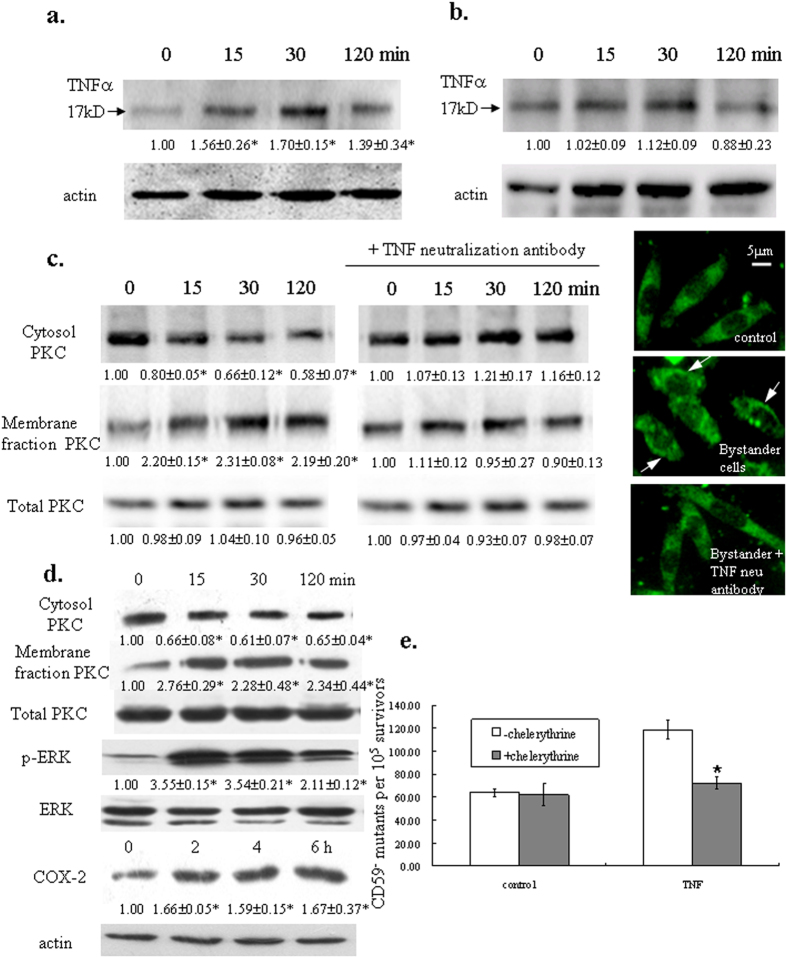
TNF alpha released by irradiated cells caused translocation of PKC and the related bystander effect. TNFα levels in irradiated (**a**) and bystander cells (**b**). Proteins from directly irradiated or bystander cells were isolated and probed with TNFα antibody. (**c**) Neutralization of TNFα suppressed PKC translocation in bystander cells. 30 ng/ml TNFα neutralization antibody was added 1 h before cells were subjected to irradiation. Membrane fraction proteins were isolated and detected with PKCα antibody. (**d**) TNFα caused translocation of PKC and resulted in ERK activation and COX-2 up-regulation. Cells were treated with 10 ng/ml TNFα for the indicated time period. Proteins were then isolated as described before and subjected to western blotting. For all western blottings, three independent experiments were performed and a representative blot was shown. Ratios of the corresponding band intensity compared with that of untreated control were measured and calculated with Image J and indicated under each band. Asterisk indicates significant difference between untreated control and the treated groups (*p* < 0.05). (**e**) Inhibition of PKC translocation suppressed TNFα-induced mutagenic effect. Exponentially growing A_L_ cells were treated with TNFα alone or in combination with chelerythrine chloride. Data are pooled from three independent experiments. Bars indicate ± S.D. of means. Asterisk indicates significant difference between chelerythrine chloride treatment and the untreated control (*p* = 0.0003).

Tumor necrosis factor α (TNFα) is a pleiotropic cytokine that mediates various biological responses in different cell lines. TNFα can inhibit cell growth, induce differentiation and apoptosis, modify gene expression and activate protein phosphorylation pathways. It was suggested that PKC translocation may play an important role in TNF signal transduction in Jurkat, K562 and U937 cells^26^. In directly irradiated A_L_ cells, we observed an increase in mature TNFα (17KD) level 15 min after α particle irradiation, the increase sustained for at least 30 min, and decreased at the 2 hr time point (Fig. 2a). However, there was no significant change in TNFα level in bystander A_L_ cells over the time frame we investigated (Fig. 2b).To further evaluated the relationship of TNFα and PKC translocation in radiation-induced bystander effect, we used TNFα neutralization antibody to pre-treat cells and analyzed PKC distribution after irradiation. As shown in Fig. 2c, blockage of the TNFα effect resulted in inhibition of PKC translocation. Activation of ERK and the up-regulation of COX-2 were suppressed by TNFα neutralization antibody as well (Supplementary Fig. S2). On the other hand, when A_L_ cells were subjected to TNF alpha treatment, PKC was observed to accumulate along cell membrane. ERK was activated and COX-2 expression was up-regulated as well (Fig. 2d). In addition, mutagenic yield of A_L_ cells was significantly increased after TNFα treatment but was attenuated by pre-treatment of PKC translocation inhibitor chelerythrine chloride (Fig. 2e). These results suggested that elevated TNFα level in the irradiation system may be responsible for the re-distribution of PKCα and activation of the downstream pathways.

### TNFR1 level was increased in bystander cells

**Figure 3:**
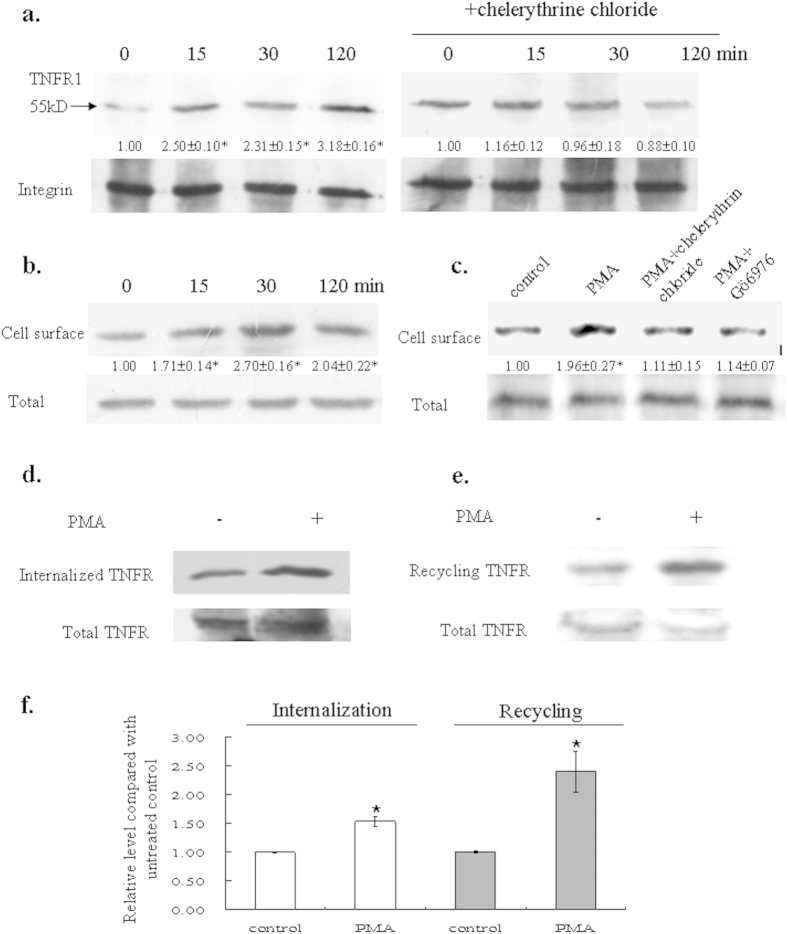
PKC activation affected TNFR1 distribution within A_L_ cells. (a) TNFR1 expression in bystander cells. Membrane fraction of the bystander cells was isolated, subjected to western blotting and probed with TNFR1 antibody. Integrin was used as loading control. (**b**) Accumulation of TNFR1 in membrane fraction after PKC activation. Cells were treated with 1 μM PKC activator PMA and analyzed as described before. (**c**) PKC inhibitors suppressed TNFR1 accumulation along cell membrane. Cells were treated with 1 μM PMA for 30 min without or with PKC inhibitors chelerythrine chloride (10 μM) or Gö6976 (5 nM). For all western blottings, three independent experiments were performed and a representative blot was shown. Ratios of the corresponding band intensity compared with that of untreated control were measured and calculated with Image J and indicated under each band. Asterisk indicates significant difference between untreated control and the treated groups (*p* < 0.05). (**d,e**) PMA accelerated internalization and recycling of TNFR1. Cells were labeled with biotin, then treated without or with 1 μM PMA for 30 min at 37 °C for protein internalization and recycling as described in the “Methods” section. Biotin-labeled proteins were pull-downed by streptavidin beads, separated by SDS-PAGE, followed by western blotting. (**f** ) Relative level of internalized and recycled TNFR1 after PKC activation. Data are pooled from three independent experiments. Bars indicate ± S.D. of means. Asterisk indicates significant difference between PMA treatment and the untreated control (*p* = 0.004 for internalization and *p* = 0.0005 for recycling).

Cellular response to TNFα is mediated through interaction with two TNF receptors TNFR1 and TNFR2. TNFR1 is expressed in various tissues and cytotoxicity elicited by TNF mostly acts through TNFR1; while TNFR2 is typically found in cells of the immune system, and mainly responds to the membrane-bound form of TNFα^27^,^28^. We therefore examined the expression of TNFR1 in bystander A_L_ cells and found an increased level of TNFR1 in the membrane fraction (Fig. 3a, left panel). We further performed biotinylation labeling and western blotting to confirm the location of accumulated TNFR1 and found out that the receptor was accumulated on plasma membrane. When cells were pretreated with chelerythrine chloride before they were subjected to irradiation, suppression of such an increment was observed (Fig. 3a, right panel), suggesting a link between PKC translocation and the accumulation of TNFR1 along the cell membrane. We next wanted to see whether the accumulation of TNFR1 in A_L_ cell surface was due to PKC activation. When A_L_ cells were treated with PMA, an activator of PKC, PKC level was elevated in the membrane fraction (Supplementary Fig. S3). The level of TNFR1 was increased as well (Fig. 3b). When cells were co-treated with PMA and PKC inhibitors chelerythrine chloride or Gö6976, the elevation of TNFR1 on cell membrane was attenuated (Fig. 3c), implicating that activation of PKC may increase TNFR1 level on plasma membrane.

To further investigate the role of PKC in the elevated TNFR1 level, we applied a method previously utilized by us and others^29^,^30^ to examine the internalization and recycling of TNFR1 when PKC was activated by PMA. As shown in Fig. 3d,e, PMA increased both internalization and recycling of TNFR1. However, the effect on recycling was more significant than on TNFR1 internalization (*p* = 0.008), which resulted in a higher amount of the receptor present on cell surface after activation of PKC (Fig. 3f).

## Discussion

PKCs can be activated by many extracellular signals and in turn modify the activities of downstream cellular proteins such as receptors, enzymes, cytoskeletal proteins, and transcription factors^31^. Upon stimulation, PKCα can be translocated from cytosol to plasma membrane, nuclei, focal adhesions, and regions of cell-cell contact^32^. Our results showed that PKC translocated from cytosol to cell membrane in bystander cells. The translocation of PKC occurred as early as 15 min after irradiation and declined at 2 hr post irradiation, suggesting PKC translocation may occur as a relative early event in the radiation-induced bystander response. In bystander cells, exogenous signals from irradiated cells are first perceived on the cell membrane, it is therefore likely that regulatory proteins such as PKCα translocates to plasma membrane in response to these signals and subsequently activate a series of downstream pathways. Indeed, our study showed that inhibition of PKC translocation with chelerythrine chloride led to suppression of ERK activity and COX-2 up-regulation as well as reduction of mutagenic yield in bystander cells. It was demonstrated that COX-2 signaling pathway plays an important role in the bystander process, and the activation of MAPK pathways is essential for the induction of COX-2^10^. The inhibitory effect of chelerythrine chloride on ERK and COX-2 implicated that PKC may act upstream of the ERK/MAPK cascade, suggesting that translocation of PKC serves as an early response in bystander effects.

TNFα has been implicated in various cellular functions such as apoptosis, proliferation, survival, and differentiation^33^. Our present study showed that over the time frame examined, TNFα level only increased in directly irradiated but not in bystander cells, though this does not rule out the possibility that TNFα may elevate at a later time point, when related pathways are activated in bystander cells. We found that TNFα activated the translocation of PKC, which implicated that increased level of TNFα in the irradiated system may be involved in the re-distribution of PKC. Indeed, when A_L_ cells were pre-treated with TNFα neutralizatoin antibody, PKC translocation in bystander cells was suppressed. On the other hand, we also found that PKC inhibitor chelerythrine chloride had an inhibitory effect on TNFR1 expression in the membrane fraction of bystander cells. These results suggested that PKC translocation may play a role in the increased expression of TNFR1 in cell membrane. Further investigation revealed that PKC activation accelerated both internalization and recycling of TNFR1, though its effect on the receptor recycling was much more significant and thus may explain the increased expression of TNFR1 on the plasma membrane of bystander cells.

**Figure 4:**
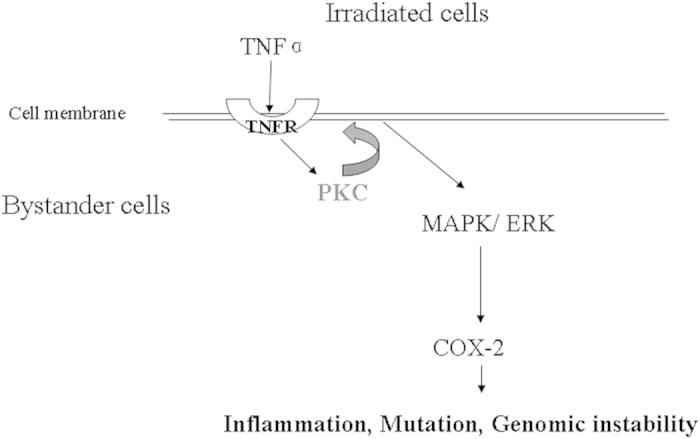
Proposed scheme of PKC-mediated pathway in bystander responses.

Although bystander effect has been well documented over the past decade, its underlying mechanism is still poorly understood. Here we focused our study within an early time frame after irradiation, and found out that within 15 min, PKC translocated from cytosol to membrane fraction in bystander cells, possibly in response to the increased level of TNFα in the irradiated system. In turn, the translocation (and thus activation) of PKC accelerated the internalization and recycling of TNFR1, which resulted in increased amount of the TNFR1 present on the cell surface. These events thus enable bystander cells to perceive signals secreted by directly irradiated cells and subsequently lead to activation of ERK and elevated COX-2 expression (Fig. 4). Our current data suggested a critical role of PKC in bystander responses, especially at early time points after irradiation. The identification of PKC as a positive mediator for the TNFα induced signaling pathways helps us better understand the molecular and cellular mechanisms of radiation-induced bystander effect.

## Methods

### Cell culture

The human-hamster hybrid A_L_ cell line which contains a standard set of CHO-K1 chromosomes and a single copy of human chromosome 11 was used^34^. The *CD59* (also known as *M1C1*) gene is located at 11 p 13.5, which encodes the CD59 cell-surface antigen marker (formerly known as S1) that renders A_L_ cells sensitive to killing by the monoclonal antibody E7 in the presence of rabbit serum complement (EMD Biosciences, Inc., La Jolla, CA). Antibody specific to the CD59 antigen was produced from a hybridoma culture. Cells were cultured in Ham’s F12 medium supplemented with 8% heat-inactivated fetal bovine serum (Invitrogen, Carlsbad, CA), 200 μM glycine, and 25 μg/ml gentamycin at 37 °C in a humidified 5% CO_2_ incubator. All the chemicals are from Sigma (St. Louis, MO) except where otherwise indicated. All the antibodies used for western blotting are from Beyotime Biotechnology (Jiangsu, China) except otherwise indicated.

### Irradiation Procedure

Radiation was carried out with an alpha particle emitter assembled at Hefei Institute of Physical Science, Chinese Academy of Sciences. The emitter delivers α particles derived from ^241^Am (with a source energy of 5.48 MeV and attenuated by a 2 cm air column to a peak energy of 3.5 MeV) at a dose rate of 1 cGy per second^35^. Exponentially growing A_L_ cells were plated on a 3-μm-thick mylar sheet 24 h before irradiation. Half of the plate was wrapped with aluminum foil before irradiation. Because the α particles can not penetrate through the aluminum foil, cells grown on this section of the plate would effectively become the bystander cells, being seeded right next to the cells that were directly irradiated. A total of 50-cGy dose was delivered to the cells. After irradiation, at different time points, cells were washed three times with cold phosphate-buffered saline (PBS), the mylar sheet was removed from the dish, cut in half along where aluminum foil was wrapped to separate the bystander cells from directly irradiated cells. Cells on the mylar sheet were then gently scraped down with a plastic scraper, and collected for further analysis.

### Western Blotting

Proteins were extracted from either irradiated or bystander cells by RIPA buffer (50 mM Tris, 150 mM NaCl, 0.1% SDS, 1% NP-40, protease inhibitors phenylmethylsulfonyl fluoride, 200 μg/ml, leupeptin, 3 μg/ml, pH 7.4) or membrane fraction isolation buffer (250 mM sucrose, 20 mM HEPES, 1.5 mM MgCl_2_, 1 mM EDTA, pH 7.4, protease inhibitors phenylmethylsulfonyl fluoride, 200 μg/ml, leupeptin, 3 μg/ml) and subjected to sonication with a Branson S450-D digital sonifier (Branson Ultrasonic, Danbury, CT). Protein concentrations were determined by Bio-Rad Protein Assay (Bio-Rad, Hercules, CA). Equivalent amounts of protein were separated on a 7.5% SDS polyacrylamide gel, transferred to a polyvinylidene difluoride (PVDF) membranes (Millipore, Billerica, MA), and incubated with different antibodies. The band intensities were evaluated by Image J and normalized to the expression level of loading controls.

### Cell surface biotinylation

Cell surface expression level of TNFR was examined using the membrane-impermeable biotinylation reagent NHS-SS-biotin as described before^36^. In brief, cells in 6-well plate were labeled with NHS-SS-biotin, lysed with RIPA lysis buffer and cell debris was removed by centrifugation. Streptavidin-agarose beads were added to the supernatant to bind the biotin-labeled proteins. Bound proteins were then released in Laemmli sample buffer (50 mM Tris-HCl pH 6.8, 2% SDS, 10% glycerol, 2.5% β-mercaptoethanol and 0.02% bromophenol blue), loaded onto a 7.5% SDS-polyacrylamide gel, separated by electrophoresis, then transferred to a PVDF membrane and detected with anti-TNFR1 antibody (1:1000 dilution, Abcam, Cambridge, UK).

### Immunofluorescence of A_L_ Cells

Cells were washed three times with PBS, fixed for 20 min at room temperature in 4% (w/v) paraformaldehyde in PBS and washed again with PBS. The fixed cells were permeabilized with 0.1% Triton X-100 for 5 min. Cells were then incubated for 1 hr at room temperature in PBS containing 1% (v/v) bovine serum albumin, after which were incubated overnight at 4 °C in the same medium containing anti-PKCα antibody. The cells were washed and bound primary antibodies were detected by reaction with Alexa Fluor488 goat anti-rabbit IgG (Invitrogen, Carlsbad, CA), diluted 1:1000, for 1 h. Cells were thoroughly washed and the mylar stripe were mounted in Fluoromount mounting medium. Samples were examined using an Olympus IX83 inverted fluorescence microscope (Olympus Corporation, Tokyo, Japan).

### Mutation assay

To determine the mutant yield, after a seven-day expression period, 5 × 10^4^ cells were plated into each of six 60 mm dishes in a total of 2 ml growth medium as described^37^. The cells were incubated in incubator for 2 hrs to allow cell attachment. Then 3% (v/v) CD59 antiserum and 1.6% (v/v) freshly thawed complement were added to each dish. The cultures were further incubated for 7–10 days before they were fixed, stained and scored for the number of CD59^−^ mutants. The cultures from each treatment were tested for mutant yield for two consecutive weeks to ensure the full expression of the mutation. Mutant fractions were calculated as the number of surviving colonies divided by the total number of cells plated after correction for non-specific killing due to complement alone.

### Internalization and recycling assay

Analysis for TNFR internalization and recycling after PKC activation was performed as described before^29^. Briefly, for internalization, monolayer cells were labeled with 1.0 mg/ml NHS-SS-biotin for 30 min. Then cells were rapidly warmed up to37 °C by pre-warmed PBS with or without PMA (1 μM). Internalization was stopped at 30 min and residual biotin on the cell surface was stripped off by incubating cells with 50 mM sodium 2-mercaptoethanesulfonate (MesNa) in NT buffer (150 mM NaCl, 1 mM EDTA, 0.2% bovine serum albumin, 20 mM Tris, pH 8.6). MesNa is a nonpermeant reducing agent that cleaves disulfide bond and thus liberates biotin from biotinylated proteins on the cell surface. The cells were then dissolved on ice for 1 h in RIPA buffer. After cell debris was removed by centrifugation, thirty microliters of cell lysate was transferred to a clean eppendorf tube and would be used as a TNFR1 total protein control. Streptavidin- agarose beads were added to the remaining cell lysate to bind the intracellular biotin-labeled proteins. Protein was then analyzed as described before. In the recycling experiments, cells were first biotinylated with 1.0 mg/ml NHS-SS-biotin at 4 °C for 30 min and then replaced with new NHS-SS-biotin (1.0 mg/ml, 37 °C). Biotinylation was stopped at 30 min and biotinylated proteins were subsequently pulled-down by streptavidin beads and analyzed as described above.

### Statistical Analysis

Data were calculated as means and standard deviations. Comparisons were made by the Student’s t tests. Differences between means are regarded as significant if *p* < 0.05.

## Additional Information

**How to cite this article**: Fang, Z. *et al.* The role of protein kinase C alpha translocation in radiation-induced bystander effect. *Sci. Rep.* **6**, 25817; doi: 10.1038/srep25817 (2016).

## Supplementary Material

Supplementary Information

## References

[b1] Morgan W. F. Non-targeted and delayed effects of exposure to ionizing radiation: I. Radiation-induced genomic instability and bystander effects *in vitro*. Radiat Res 159, 567–580 (2003). 12710868 10.1667/0033-7587(2003)159[0567:nadeoe]2.0.co;2

[b2] Little J. B. Genomic instability and bystander effects: a historical perspective. Oncogene 22, 6978–6987 (2003). 14557801 10.1038/sj.onc.1206988

[b3] Hei T. K. *et al.* Mechanism of radiation-induced bystander effects: a unifying model. J Pharm Pharmacol 60, 943–950 (2008). 18644187 10.1211/jpp.60.8.0001 PMC4410683

[b4] Mancuso M. *et al.* Oncogenic bystander radiation effects in patched heterozygous mouse cerebellum. Proc Natl Acad Sci USA 105, 12445–12450 (2008). 18711141 10.1073/pnas.0804186105 PMC2517601

[b5] Azzam E. I., de Toledo S. M. & Little J. B. Direct evidence for the participation of gap junction-mediated intercellular communication in the transmission of damage signals from alpha -particle irradiated to nonirradiated cells. Proc Natl Acad Sci USA 98, 473–478 (2001). 10.1073/pnas.011417098 PMC14611 11149936

[b6] Zhou H. *et al.* Radiation risk to low fluences of alpha particles may be greater than we thought. Proc Natl Acad Sci USA 98, 14410–14415 (2001). 10.1073/pnas.251524798 PMC64695 11734643

[b7] Mothersill C. & Seymour C. B. Cell-cell contact during gamma irradiation is not required to induce a bystander effect in normal human keratinocytes: evidence for release during irradiation of a signal controlling survival into the medium. Radiat Res 149, 256–262 (1998). 9496888

[b8] Iyer R., Lehnert. B. E. & Svensson R. Factors underlying the cell growth-related bystander responses to alpha particles. Cancer Res 60, 1290–1298 (2000). 10728689

[b9] Matsumoto H. *et al.* Induction of radioresistance by a nitric oxide-mediated bystander effect. Radiat. Res. 155, 387–396 (2001). 11182788 10.1667/0033-7587(2001)155[0387:iorban]2.0.co;2

[b10] Zhou H. *et al.* Mechanism of radiation-induced bystander effect: role of the cyclooxygenase-2 signalling pathway. Proc Natl Acad Sci USA 102, 14641–14646 (2005). 16203985 10.1073/pnas.0505473102 PMC1253564

[b11] Zhou H., Ivanov V. N., Lien Y. C., Davidson M. & Hei T. K. Mitochondrial function and nuclear factor-kappaB-mediated signalling in radiation-induced bystander effects. Cancer Res 68, 2233–2240 (2008). 10.1158/0008-5472.CAN-07-5278 PMC3715144 18381429

[b12] Ivanov V. N. *et al.* Radiation-induced bystander signalling pathways in human fibroblasts: a role for interleukin-33 in the signal transmission. Cell Signal 22, 1076–1087 (2001). 10.1016/j.cellsig.2010.02.010 PMC2860693 20206688

[b13] Ghandhi S. A., Ming L., Ivanov V. N., Hei T. K. & Amundson S. A. Regulation of early signalling and gene expression in the alphaparticle and bystander response of IMR-90 human fibroblasts. BMC Med Genomics 3, 31 (2010). 20670442 10.1186/1755-8794-3-31 PMC2919438

[b14] Fima E. *et al.* PKC-η enhances cell cycle progression, the expression of G1 cyclins and p21 in MCF-7 cells. Oncogene 46, 6794–6804 (2001). 11709714 10.1038/sj.onc.1204885

[b15] Olson E. N., Burgess R. & Staudinger J. Protein kinase C as a transducer of nuclear signals. Cell Growth Differ 4, 699–705 (1993). 8398911

[b16] Claro S., Kanashiro C. A., Oshiro M. E., Ferreira A. T. & Khalil R. A. alpha- and epsilon-protein kinase C activity during smooth muscle cell apoptosis in response to gamma-radiation. J Pharmacol Exp Ther. 322, 964–972 (2007). 17600140 10.1124/jpet.107.125930

[b17] Lee S. J. *et al.* Adaptive response is differently induced depending on the sensitivity to radiation-induced cell death in mouse epidermal cells. Cell Biol Toxicol 16, 175–184 (2000). 11032361 10.1023/a:1007658905639

[b18] Nakajima T. & Yukawa O. Radiation-induced translocation of protein kinase C through membrane lipid peroxidation in primary cultured rat hepatocytes. Int J Radiat Biol 70, 473–480 (1996). 8862459 10.1080/095530096144950

[b19] Choi E. K. *et al.* Effect of protein kinase C inhibitor (PKCI) on radiation sensitivity and c-fos transcription. Int J Radiat Oncol Biol Phys 49, 397–405 (2001). 11173133 10.1016/s0360-3016(00)01485-1

[b20] Baskar R., Balajee A. S., Geard C. R. & Hande M. P. Isoform-specific activation of protein kinase c in irradiated human fibroblasts and their bystander cells. Int J Biochem Cell Biol 40, 125–134 (2008). 17709275 10.1016/j.biocel.2007.07.002

[b21] Newton A. C. Protein kinase C: structure, function, and regulation. J Biol Chem 270, 28495–28498 (1995). 7499357 10.1074/jbc.270.48.28495

[b22] Loegering D. J. & Lennartz M. R. Protein kinase C and toll-like receptor signaling. Enzyme Res 2011, 537821 (2011). 21876792 10.4061/2011/537821 PMC3162977

[b23] Lee S. J., Cho C. K., Yoo S. Y., Kim T. H. & Lee Y. S. Influence of ionizing radiation on induction of apoptotic cell death and cellular redistribution of protein kinase C isozymes in mouse epidermal cells differing in carcinogenesis stages. Mutat Res 426, 41–49 (1999). 10320749 10.1016/s0027-5107(99)00078-0

[b24] Chao M. D., Chen I. S. & Cheng J. T. Inhibition of protein kinase C translocation from cytosol to membrane by chelerythrine. Planta Med 64, 662–663 (1998). 9810275 10.1055/s-2006-957545

[b25] Dent P., Yacoub A., Fisher P. B., Hagan M. P. & Grant S. MAPK pathways in radiation responses Oncogene 22, 5885–58896 (2003). 12947395 10.1038/sj.onc.1206701

[b26] Schütze S., Nottrott S., Pfizenmaier K. & Krönke M. Tumor necrosis factor signal transduction. Cell-type-specific activation and translocation of protein kinase C. J Immunol 144, 2604–2608 (1990). 2156927

[b27] Tartaglia L. A., Rothe M., Hu Y. F. & Goeddel D. V. Tumor necrosis factor’s cytotoxic activity is signaled by the p55 TNF receptor. Cell 73, 213–216 (1993). 8386591 10.1016/0092-8674(93)90222-c

[b28] Wajant H., Pfizenmaier K. & Scheurich P. Tumor necrosis factor signaling. Cell Death Differ. 10, 45–65 (2003). 12655295 10.1038/sj.cdd.4401189

[b29] Hong M., Hong W., Ni C., Huang J. & Zhou C. Protein kinase C affects the internalization and recycling of organic anion transporting polypeptide 1B1. Biochim Biophys Acta 1848, 2022–2030 (2015). 26009271 10.1016/j.bbamem.2015.05.011

[b30] Zhang Q. *et al.* Organic anion transporter OAT1 undergoes constitutive and protein kinase C-regulated trafficking through a dynamin- and clathrin-dependent pathway. J Biol Chem 283, 32570–32579 (2008). 18818201 10.1074/jbc.M800298200 PMC2583290

[b31] Breitkreutz D., Braiman-Wiksman L., Daum N., Denning M. F. & Tennenbaum T. Protein kinase C family: on the crossroads of cell signaling in skin and tumor epithelium. J Cancer Res Clin Oncol 133, 793–808 (2007). 17661083 10.1007/s00432-007-0280-3 PMC12160839

[b32] Nakashima S. Protein kinase C alpha (PKC alpha): regulation and biological function. J Biochem 132, 669–675 (2002). 12417014 10.1093/oxfordjournals.jbchem.a003272

[b33] Gaur U. & Aggarwal B. B. Regulation of proliferation, survival and apoptosis by members of the TNF superfamily. Biochem Pharmacol 66, 1403–1408 (2003). 14555214 10.1016/s0006-2952(03)00490-8

[b34] Waldren C. A., Jones C. & Puck T. T. Measurement of mutagenesis in mammalian cells. Proc Natl Acad Sci USA 76, 1358–1362 (1979). 286318 10.1073/pnas.76.3.1358 PMC383250

[b35] Chen N., Wu L., Yuan H. & Wang J. ROS/Autophagy/Nrf2 pathway mediated low-dose radiation induced radio-resistance in human lung adenocarcinoma A549 Cell. Int J Biol Sci 11, 833–844 (2015). 26078725 10.7150/ijbs.10564 PMC4466464

[b36] Li N. *et al.* Identification of amino acids essential for estrone-3-sulfate transport within transmembrane domain 2 of organic anion transporting polypeptide 1B1. PLoS One 7, e36647 (2012). 22574206 10.1371/journal.pone.0036647 PMC3344916

[b37] Hei T. K., Liu S. X. & Waldren C. A. Mutagenicity of arsenic in mammalian cells: role of reactive oxygen species. Proc Natl Acad Sci USA 95, 8103–8107 (1998). 9653147 10.1073/pnas.95.14.8103 PMC20936

