# Supplementary material for: The role of protein kinase C alpha translocation in radiation-induced bystander effect
Source: Sci Rep. 2016 May 11;6:25817. doi: 10.1038/srep25817 (PMC4863171; doi:10.1038/srep25817)
Supplement: Supplementary Information [file srep25817-s1.pdf]

Supplemental Figure S1 Radiation-induced bystander effect inhibited by PKCα inhibitor Gö6976

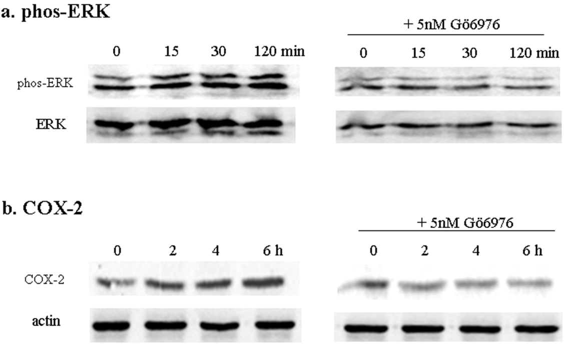

Bystander cells were treated with selective PKCα inhibitor Gö6976. Gö6976 is a potent and selective PKC inhibitor for conventional PKC isoforms PKCα and β1, with IC50 values of 2.3nM and 6.2 nM, respectively. We therefore used a concentration of 5nM to differentiate the effect caused by PKC α or β1. Proteins were extracted, separated with SDS-PAGE, transferred to PVDF membrane and probed with different antibodies. Three independent experiments were performed and a representative blot was shown.

Supplemental Figure S2 TNFα neutralization antibody suppressed activation of ERK and up-regulation of COX-2

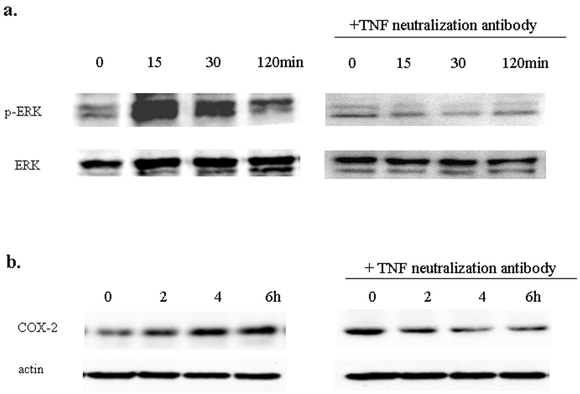

30ng/ml TNF α neutralization antibody was added 1h before cells were subjected to irradiation. Bystander cells were collected at time points indicated and proteins were extracted, separated with SDS-PAGE, transferred to PVDF membrane and probed with ERK or COX-2 antibodies. Three independent experiments were performed and a representative blot was shown.

Supplemental Figure S3 PMA affect cellular distribution of PKCα

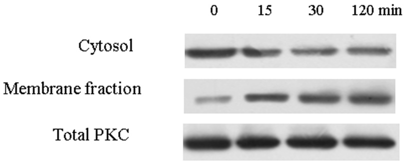

Cells were treated with 1μM PKC activator PMA and collected at different time points. Membrane proteins were extracted, separated with SDS-PAGE, transferred to PVDF membrane and probed with PKCα antibody. Three independent experiments were performed and a representative blot was shown.
